# Body Composition in International Sprint Swimmers: Are There Any Relations with Performance?

**DOI:** 10.3390/ijerph17249464

**Published:** 2020-12-17

**Authors:** Milivoj Dopsaj, Ilona Judita Zuoziene, Radoje Milić, Evgeni Cherepov, Vadim Erlikh, Nerijus Masiulis, Andrea di Nino, Janez Vodičar

**Affiliations:** 1 Institute of Sport, Tourism and Service, South Ural State University, 454080 Chelyabinsk, Russia; cherepovea@susu.ru (E.C.); erlikhvv@susu.ru (V.E.); 2 Faculty of Sport and Physical Education, University of Belgrade, Blagoja Parovića ul. 156, 11000 Belgrade, Serbia; 3 Faculty of Sports Biomedicine, Lithuanian Sports University, Sporto str. 6, LT-44221 Kaunas, Lithuania; Ilona.Zuoziene@lsu.lt (I.J.Z.); Nerijus.Masiulis@lsu.lt (N.M.); 4 Faculty of Sport, University of Ljubljana, Gortanova ul. 22, 1000 Ljubljana, Slovenia; phycenter@gmail.com (R.M.); Janez.Vodicar@fsp.uni-lj.si (J.V.); 5 ADN Swim Project, 81100 Caserta, Italy; adinino@gmail.com

**Keywords:** sprint swimmers, body composition, results prediction, body fat, skeletal muscle mass

## Abstract

The paper addresses relations between the characteristics of body composition in international sprint swimmers and sprint performance. The research included 82 swimmers of international level (N = 46 male and N = 36 female athletes) from 8 countries. We measured body composition using multifrequency bioelectrical impedance methods with “InBody 720” device. In the case of male swimmers, it was established that the most important statistically significant correlation with sprint performance is seen in variables, which define the quantitative relationship between their fat and muscle with the contractile potential of the body (Protein-Fat Index, r = 0.392, *p* = 0.007; Index of Body Composition, r = 0.392, *p* = 0.007; Percent of Skeletal Muscle Mass, r = 0.392, *p* = 0.016). In the case of female athletes, statistically significant relations with sprint performance were established for variables that define the absolute and relative amount of a contractile component in the body, but also with the variables that define the structure of body fat characteristics (Percent of Skeletal Muscle Mass, r = 0.732, *p* = 0.000; Free Fat Mass, r = 0.702, *p* = 0.000; Fat Mass Index, r = −0.642, *p* = 0.000; Percent of Body Fat, r = −0.621, *p* = 0.000). Using Multiple Regression Analysis, we managed to predict swimming performance of sprint swimmers with the help of body composition variables, where the models defined explained 35.1 and 75.1% of the mutual variability of performance, for male and female swimmers, respectively. This data clearly demonstrate the importance of body composition control in sprint swimmers as a valuable method for monitoring the efficiency of body adaptation to training process in order to optimize competitive performance.

## 1. Introduction

A sports training system represents a long-lasting and multicomplex process in which programmed training loads are applied. It is well documented in scientific literature that has examined long-term athlete development that selected athletes need eight to twelve years of systematic sports training with approximately two or three hours of daily practice to achieve an elite level with full performance potential [1,2].

The improvement of the sports training system has led to better sport achievements with the help of increased athlete performance. The same phenomena were established in swimming in the last 50 years, regardless of swimmers’ age and gender [3].

Biologically, development of athletes as a specifically selected and highly trained population, is constantly subjected to adaptation. It is well known that there are many extraneous factors (genetics, anatomical, neurological, hormonal, psychological, cognitive etc.) that must be incorporated within the planning of any specific form of physical training. All these systems are influenced by the hetero-chronic phenomenon, that is, they are time independent in their biological development, but all of them affect the physiological systems of the body [4].

One of the mechanisms of sports training is the morphological adaptation of the body. Adaptation in sports is always triggered by training and training components, such as the type of intensity or training load, which is aimed at changing the morphological characteristics of the body to reach a typical body structure [4,5,6,7,8,9]. This phenomenon is only occurring to those body tissues that are subjected to biological adaptation, as are fat and muscle tissue or even a bone tissue component [9,10,11].

The published evidence on morphological factors which contributes to the development of elite performance in swimming, has not been represented sufficiently in top scientific literature in the previous 35 years [11,12,13,14,15]. Competitive swimmers, especially Olympic elite swimmers, were found to be taller than sub-Olympic. The longitudinal data from Russian national swimming teams demonstrated that current swimmers tend to be taller than in the past. For example, 17-year-old male swimmers have an average body height of more than 185 cm [16].

In swimming, as a form of human locomotion in water, as a physical medium, metabolic power (the energy expended in the unit of time) exponentially increase as a function of speed (as a “measure” of exercise intensity). The maximum swimming speed will be achieved by the swimmer who can achieve higher maximum metabolic power for lower energy consumption during swimming [17]. The overall efficiency of swimming depends on the propulsive efficiency and the hydrodynamic resistance (different aspects of passive and active drag form) that the body creates as it moves through the water. Different forms of water resistance, as a sum of overall drag, during swimming is affected by the swimmer’s body shape and morphology, body density and body position in a water—as passive drag components; or depends on lots of comprehensive factors such as friction drag, frontal area and body shape resistance drag, and wave drag—as active drag components [15,18]. To enable an athlete to swim faster, the swimmer must either increase the propulsive force or reduce the drag force, or, ideally, do both [17,19,20].

Body composition is a term that describes the relative proportions of all main components of the body, including fat, bone, muscle, and water mass [21]. In the last decade, bioelectrical impedance analysis (BIA), especially direct segmental multifrequency methods, have been widely used in science and sport practice—along with the other traditional body composition methods as skinfold measurements, dual-energy X-ray absorptiometry, body density measurement, and total body water estimates—and had become a standard method for determination of complete body structure according to the body segments [7,22,23,24].

Evaluating the relationship of body composition to swim performance on the sample of 280 competitive female swimmers it was found that Body Height, Body Mass, Lean Body Mass (LBM), and Residual Lung Volume (RV) were in correlation, which is statistically significant but inverse to the time recorded for the 100-yard swim [25]. During competitive swim season, significant increase of LBM and decrease of fat mass has occurred primarily during the part of the season when training was intense [12]. Increasing muscle size and improvement of muscle contractile quality, and decreasing fat mass, at a level of optimal balance, may have positive effect on swimming performance, and is likely to be important for maximizing competitive swimming performance. Moreover, seasonal swim performance changes tend to follow changes in muscle mass, so regular control of body composition during the season may be helpful and beneficial in order to improvement training and performance [11].

This research is aimed at defining the relations between swimming performance and the characteristics of body composition in international sprint swimmers, measured with the multifrequency bio-impedance method. The secondary goal of the research is to define a multidimensional model of performance prediction, based on the most sensitive variables of body composition in swimmers. This model can serve as a tool for controlling, achieving, and maintaining optimal body composition of sprinter swimmers.

All the results obtained in this research may be used to improve sports training technologies in sprint swimming disciplines in the athletes of an international level.

## 2. Materials and Methods

This research was conducted using Cross-Sectional Designs, applied according to the research methods accepted for physical activity and sport [26]. Moreover, in this study, we used a multicentric study design and laboratory testing method. This study possesses the characteristics of fundamental and applied research, aimed at expanding the existing knowledge about the body composition of international swimmers in relation to sprint performance potential. Sprint performance has been expressed as FINA point score (Federatioin Internationale de Natation, http://www.fina.org/content/fina-points), where FINA points score allows standardized comparisons of swimming performances in different events.

### 2.1. Research Sample

Eighty-two swimmers of an international level (N = 46 male athletes, age—22.9 ± 4.2 years, training experience—14.60 ± 5.6 years, FINA Score = 785 ± 71, Min = 638–Max = 883 and N = 36 female swimmers, FINA Score = 727 ± 98, Min = 642–Max = 910, age—21.0 ± 4.7 years, training experience—12.7 ± 4.6 years) participated in this research. The participants were from the following 8 countries: Serbia (N = 33, 20 males and 13 females), Slovenia (N = 23, 9 males and 14 females), Lithuania (N = 10 males), Russia (N = 5 males), Estonia (N = 3 females), Belarus (N = 3 females), and Bosnia and Herzegovina (N = 3 females). Participants used the following stroke styles: 32 freestyle (15 males and 17 females), 14 backstroke (5 males and 9 females), 20 breaststroke (16 males and 4 females), and 14 fly swimmers (10 males and 6 females). All swimmers were competitors at the distance of 50 m or 100 m.

### 2.2. Measurement Procedure

Before testing, the participants, all of them volunteers, were informed about measurement conditions and procedures. The overall process of sample tracking was carried out during the period 2012–2017. The study was conducted at the premises of three scientific laboratories: the laboratory of the Faculty of Sport and Physical Education at the University of Belgrade (Serbia); the laboratory of the Faculty of Sport at the University of Ljubljana (Slovenia); and the laboratory of the Faculty of Sport Bio-medicine at Lithuanian Sports University, Kaunas (Lithuania). The research was performed in accordance with the conditions of Declaration of Helsinki: Recommendations Guiding Physicians in Biomedical Research Involving Human Subjects (http://www.cirp.org/library/ethics/helsinki/), and with the approval and consent of the Ethics Committee of all three faculties, and under the supervision of The Institutional Ethical Board of Faculty of Sport and Physical Education, University of Belgrade Serbia, approved the study (No. 484-2).

#### 2.2.1. Body Composition Variables

We measured body composition using bioelectrical impedance analysis (BIA) with InBody 720 device that used Tetapolar 8 points by tactical electrodes system with DSM-BIA (Direct Segmental Multifrequency Bioelectrical Impedance Analysis) (Biospace Co, Ltd., Seoul, Korea). Inbody 720 device [27] demonstrated high test-retest reliability and accuracy (ICC 0.9995). It is regarded to be highly statistically reliable and valid for measuring both overall and segmental body composition in female and male athletes [22,24,28].

All participants were measured in accordance with manufacturer’s suggestions and previously published procedures [7,8,27,29].

For this study, we used 14 variables, 5 of which were basic and 9 were derived (index) variables, defining the morphology and composition of the body according to the following criteria: basic component variables, voluminosity independent variables, longitudinal independent variables, and index variables.

*Basic component variables (5)*: BH—body height, cm; BM—body mass, kg; BF—body fat mass, kg; —skeletal muscle mass, kg; FFM—fat free mass, kg.

*The voluminosity independent variables (3)*: PBF—percent of body fat, calculated as: BF (body fat, kg)/BM (body mass, kg), %; PSMM—percent of skeletal muscle mass, calculated as: SMM (skeletal muscle mass, kg)/BM (body mass, kg), %; PFFM—percent of fat free mass, calculated as: FFM (fat free mass, kg)/BM (body mass, kg), %;

*Longitudinal independent variables (3)*: BMI—body mass index, calculated as: BM (body mass, kg)/BH^2^ (body height, m), kg Body mass·m^−2^; FMI—fat mass index, calculated as: FM (fat mass, kg)/BH^2^ (body height, m), kg Body fat·m^−2^; SMMI—skeletal muscle mass index, calculated as: SMM (skeletal muscle mass, kg)/BH^2^ (body height, m), kg Skeletal muscle·m^−2^; FFMI—fat free mass index, calculated as: FFM (fat free mass, kg)/BH^2^ (body height, m), kg FFM·m^−2^;

*Derivated (index) variables (2)*: PFI—protein fat index, calculated as the relation between proteins, as a pure contractile tissue in the body (kg) and body fat mass, as a ballast or non-contractile tissue in the body (kg), kg; IBC—index of body composition, calculated as the relation between BMI (kg) and the percent of body fat mass (%), arbitrary units.

All the variables used are taken from the previously published research [7,8,29,30].

#### 2.2.2. Swimming Performance Variable

The criterion variable was the value of the absolute best result at 50 m or 100 m distance in 50 m pool, achieved in a given competitive microcycle and expressed as FINA score [31]. Body composition was measured on one occasion only within a competitive microcycle, i.e., at least seven days before or after a race during a particular swimming season. Relevant body composition was compared with the actual peak of the results achieved.

### 2.3. Statistical Procedures

We processed raw results using basic descriptive statistics, namely a central tendency (MEAN), statistical dispersion (SD, cV%, Min and Max measured values), and the parameters of measurement errors. The methods used are the result of the multicentric nature of the study (SEM—Standard Error of Measurements, absolute and relative). The distribution regularity of variables was tested with the help of Kolmogorov–Smirnov nonparametric test (KSZ). We established the partial relations between criteria and body composition variables using Pearson’s correlation analysis. With the help of Fisher t-to-z transformation for an independent sample, we calculated if there were statistical differences in the correlations between male and female swimmers, having the same body composition variables and criteria, i.e., performance level. We also used a mathematical modeling by means of Multivariate Regression Analysis (MRA) as a multidimensional prediction system, which helped us to define complex relations between the criteria (swimming performance and FINA Scores) and body composition variables used as predictors [32,33]. All statistical analysis were performed using corresponding statistical software SPSS 19.0. The level of statistical significance is defined at 95% and the probability values of *p* < 0.05 [34].

## 3. Results

Table 1 demonstrates all statistical data about Male and Female swimmers, respectively. The basic anthropometrical and body composition data were shown that mean value for BH is 186.3 ± 5.4 cm, for BM is 82.4 ± 6.5 kg, for BMI is 23.73 ± 1.35 kg·m^−2^, percent of body fat (PBF) is 9.82 ± 3.35, and percent of skeletal muscle mass (PSMM) is 52.36 ± 1.83 considering male sprint swimmers sample. Female swimmers had the following basic anthropometrical and body composition mean values: BH = 173.4 ± 5.8 cm, BM = 62.8 ± 4.9 kg, BMI = 20.88 ± 1.13 kg·m^−2^, PBF = 15.79 ± 4.84, and PSMM = 47.01 ± 2.93. All variables had a normal distribution, except for one—IBC at female sample (KS *p* = 0.031), which indicates that the results can be used as acceptably representative for interpretation of the population of international sprint swimmers.

Table 2 demonstrates a correlation matrix for body composition variables and athletes’ performance with Fisher r-to-z transformation results. All statistically different r values between gender considering observed body composition variables are shown in bold format. The largest statistically important difference between individual variables of body composition in relation to the sprint swimming performance as per gender was found in PFFM (t = −2.74, *p* = 0.006) i PBF (t = 2.72, *p* = 0.007), while the smallest difference was in BF (t = 2.13, *p* = 0.033) i FFMI (t = −2.14, *p* = 0.032). In as many as five variables, it was established that the gender was not a source of influence on the performance in sprint swimming (BH, BM, BMI, IBG, and PFI, Table 2).

Table 3 demonstrates MRA statistical results with the multiple equation models of athletes’ sprint swimming performance prediction by body composition characteristics. Of those variables, which are part of the predictor system for the swimming sprint performance, the three most influential ones in males are BM, BH, and PSMM (body mass, height, and the muscle percentage) while for females those are PBF, PSMM, and PFI (the percentage of fat and muscles and the structural ratio of fat and protein). For males, 35.1% of the common variance with the criterion is explained, while 75.1% for female swimmers. Thus, there is significantly more other factors then body composition influencing sprint swimming performance in males (64.9%) than in females (24.9%).

**Figure 1:**
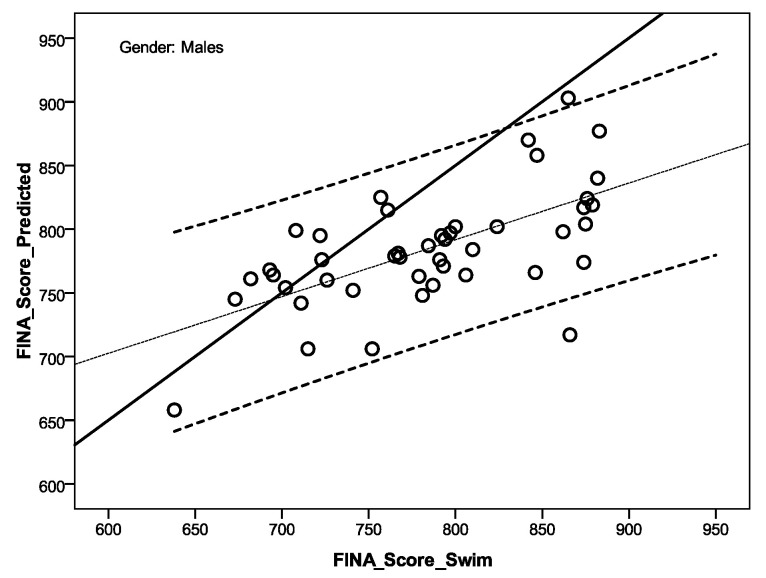
Linear regression for a calculated FINA score and FINA score performance for male sprint swimmers (y = 435.143 × FINA_Score_Swim^0.446^).

**Figure 2:**
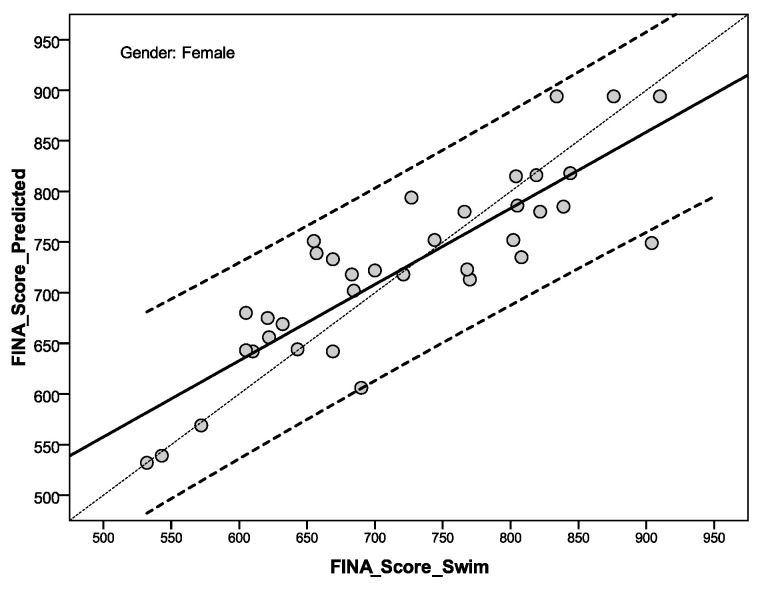
Linear regression for a calculated FINA score and FINA score performance for female sprint swimmers (y = 181.114 × FINA_Score_Swim^0.753^).

Figure 1 and Figure 2 show linear regression for a calculated FINA score (FINA_Score_Predicted) and FINA score performance (FINA_Score_Swim) for male and female sprint swimmers. On the basis of the defined multiple equation models (Table 3, performance prediction), the relations between the calculated y value (FINA_Score_Predicted), the criteria of the sprinter swimming performance (FINA_Score_Swim), and the calculated value of intercept, it is evident that for the male swimmers (Figure 1, intercept—435.143) the line of relation is slanted toward the x-axis, meaning that the model underestimates sprint performance (FINA_Score_Swim) the better the sprint performance. Namely, the better the international male swimmer’s sprint performance is, the influence of body composition on the prediction of results is lesser. Compared to international female sprint swimmers, the value of intercept is inferior; that is at the level of 118.114 (Figure 2), indicating that the better the sprint performance, the body composition characteristics will be more proportionally accurate for the sprint swimming prediction.

## 4. Discussion

The results from this cross-selection study confirmed the existence of statistically significant and complex relations between body composition characteristics and performance in international sprint swimmers, both male and female.

Averaged basic anthropo-morphological characteristics (Table 1) indicate that sprint swimmers from this sample are taller, heavier, and larger than male elite swimmers of the past (183.8 cm, 78.4 kg, and 23.21 kg·m^−2^) [35]. Female swimmers are, also, taller, have lower body weight and BMI comparing with the results published previously (171.5 cm, 63.1 kg, and 21.45 kg·m^−2^) [35]. Moreover, the results showed that the international sprint swimmers from the study, both men and women are taller, with less body fat, with lower body mass index, but with a higher level of contractile, i.e., muscle mass, than national level swimmers [30].

This statement is also supported by the results of the correlation (Table 2), on the basis of which it can be claimed that sprint performance of male swimmers have the highest level of statistically significant correlation with variables that define the relative and absolute amount of contractile, i.e., muscle tissue in the body, primarily partialized in relation to the volume of the body, in absolute value, and then in relation to longitudinal characteristics such as: PSMM (r = 0.353, *p* = 0.016), SMM (r = 0.350, *p* = 0.017), and SMMI (r = 0.323, *p* = 0.029). The second set of correlation defined index variables identify quantitative relationship, i.e., balance between fat mass and protein, and body volume and relative fat mass value such as: PFI (r = 0.392, *p* = 0.007) and IBC (r = 0.391, *p* = 0.007). Identifying the optimal balance between body characteristic as a parameters of lean mass and fat mass is likely to be beneficial and of some importance for maximizing swimming performance [11].

Comparing to the males, different correlation structure was established in female swimmers. The first set represent variables that define the absolute and relative amount of the contractile component in the body in correlation with competitive performance such as: PSMM (r = 0.732, *p* = 0.000), SMM (r = 0.730, *p* = 0.000), FFM (r = 0.702, *p* = 0.000), PFFM (r = 0.696, *p* = 0.000). However, the next set of correlations, considering their statistical significance level, is determined in terms of the structure of body fat characteristics such as: PBF (r = −0.621, *p* = 0.000), FMI (r = −0.642, *p* = 0.000), and BF (r = −0.566, *p* = 0.000). Very high statistically significant correlations for index variables were also established, identifying quantitative relationship, i.e., balance between fat mass, and body volume or protein mass: IBC (r = 0.687, *p* = 0.000) and PFI (r = 0.655, *p* = 0.000). Finally, although not found in the male section, BH, as a basic longitudinal characteristic of the body, has a statistically significant correlation with sprint swimming performance at r = 0.535 and *p* = 0.001 (Table 2).

One of the most important papers, which investigates the relation between performance, body composition, and somatotype in competitive collegiate swimmers, was published 25 years ago [14]. At the beginning of the season, the authors have found a statistically significant relation between 100-yard performance and body height (r = −0.466, *p* < 0.01), percent of body fat (r = 0.351, *p* < 0.05), and fat-free weight (r = −0.332, *p* < 0.05) only in female swimmers. At the main period of the season, they found higher correlations for the same variables of performance and body height (r = −0.766, *p* < 0.001), fat-free weight (r = −0.657, *p* < 0.001), body weight (r = −0.437, *p* < 0.01), and the ectomorphic and mesomorphic body type (r = −0.441 and 0.392, *p* < 0.05, respectively). These correlations were established only in female swimmers. Authors made a conclusion that the characteristics of body composition and somatotype may serve as performance predictors only in female athletes. The results of our study demonstrated exactly the same structure with almost the same level of correlations considering female sample (Table 2).

Results of Fisher r-to-z transformation demonstrate the existence of a statistically significant body composition variables, which influence more the female performance in comparison with the male performance (Table 2). The greatest differences between male and female characteristics (Table 2) were established between PFFM correlations (z = 2.74, *p* = 0.006), PBF (z = 2.72, *p* = 0.007) etc. The most pronounced differences were found in four variables: defining the relative values of fat-free, i.e., most likely contractile mass of the body, such as PFFM and PSMM, as a body volume independent variables (averaged *p* difference was 0.011); absolute values for the same variables, FFM and SMM (averaged *p* difference was 0.012); ballast tissue mass variables such as PBF, FMI, and BF (averaged *p* difference was 0.017); and finally, in longitudinal independent variables, defining the amount of fat-free, i.e., most likely contractile mass of the body per body high, such as FFMI and SMMI (averaged *p* difference was 0.031). These results demonstrate a hierarchical structure of gender dependent differences regarding the influence of body structure on sprint swimmers performance.

In general, it has been established (Table 2) that the greatest individual impact on the result of sprint disciplines for female swimmers is represented by body characteristics of the absolute quantity (SMM, r = 0.730, *p* = 0.000) and relative voluminosity (PSMM, r = 0.730, *p* = 0.000) of muscles in the body. Both relations are positive, which imply that for higher FINA point score (better sprint swimming performance) the higher amount muscle mass (absolute and relative) female sprint swimmers need to have.

The indicators of the ratio of ballast (fat) and contractile (protein) mass, which are absolutely related (PFI, r = 0.392, *p* = 0.007), and structurally related (IBC, r = 0.391, *p* = 0.007) have the greatest impact on the result in the sprint has the influence of body structure in males (Table 2).

According to MRA results, it is possible to predict swimming performance in international sprint swimmers with the body composition variables. The models chosen for interpretation were defined according to the criterion of least prediction error. They explained 35.1 and 75.1% of performance relation with standard error of 57.48 and 55.99 FINA score, for male and female swimmers, respectively (Table 3).

Moreover, the results of MRA models demonstrated a complex structure of the variables included in equations. In general, we used 14 variables, six and seven of them were included in equation for male and female athletes, respectively. Four variables were common for both genders (PFI, BM, PSMM, and IBC), and the rest was specific (BH and SMMI for male athletes; BMI, FFMI, and PBF for females’ athletes; Table 3).

Strength and power are highly connected with muscle size [21,36]. Thus, an increase in muscle or fat free body mass enables the athletes to produce more muscle force during specific movement efforts, which improve speed, quickness, acceleration, and agility [6,37,38,39]. Importance of body structure characteristics that strongly contribute to contractile potential of swimmers, regardless of their gender, are determined by following variables: the amount of skeletal muscle mass in the body (SMM, PSMM, and SMMI); the amount of fat-free mass in the body (FFM, PFFM, and FFMI); the structural relations between muscle and fat tissues indexes (PFI and IBC). All of these variables indicate the body potential for strength and power production during sprint swimmers. Strength parameters have been recently proposed as one of the most important specific factors that influence positively swimming performance through the mechanism of increasing stroke pulling force and stroke efficiency mechanisms regardless of age [11,20,37,40,41,42,43,44]. Therefore, the significant improvement of body strength or segmental body strength in swimmers (arms, legs, or trunk) results in a higher maximum peak force per stroke and may influence the sprint speed and turns, or may result in more stable torso during swimming [40,43,45].

Longitudinal anthropometric characteristics such as body height and the length of the upper (arm span) and lower limbs are of primary importance for achieving high results regardless the age of swimmers [13,46,47]. The results of this study have demonstrated that the equation for the prediction of swim performance recognizes basic longitudinal characteristics (BH) as an important factor independent of gender (Table 3). Morales and co-authors [46] established a fundamental fact about the importance of longitudinal characteristics in swimming. In particular, they showed that for the female and male athletes aged 9–22 and involved in 50-m freestyle swimming, the increase in swim velocity is the result of the increase in stroke length and stroke index. Of course, these parameters depend on the efficiency of stroke mechanism (hydrodynamic, energetic, and bio-mechanical dependency). However, longitudinal characteristics of the body represent anatomical and mechanical potential for this phenomenon.

The effect of the body fat variables (BF, PBF, and BFI, Table 2 and Table 3) on performance in female swimmers could be explained by body drag characteristics, as well as with active and passive drag efficiency [15,19,48]. Energy in swimming is used for maintaining the body on water surface and generating the muscle force required to overcome water resistance. Generally, swimming speed depends on the interaction of propulsive and resisting forces. Swimming efficiency and speed can improve by increasing propulsive forces and/or minimizing resisting forces that affect the body per se, or body movement at a given speed. The lower body fat probably results in lower body shape drag (frontal area) and skin friction drag, while at the same time, body composition contractile potential, which depends on variables (SMM and FFM...etc.), provides a better propulsion force potential for the faster swimming [36]. Moreover, there are significant evidence that fat reduction contributes to muscular and cardio-respiratory endurance as well as, to the development of speed and agility [11,15,21].

In the sprint swimming, it is necessary to increase stroke frequency to improve the speed of swimming. However, as that increase is not limitless because of neural coordination factors and stroke coordination and because of muscle power limitation, for faster sprint swimming, swimmers adapt their stroke technique pattern, from so-called “S-hape“ to a much more sprint-speed productive, the so-called “I-shaped“ stroke. Using that stroke style, sprint swimmers were able to continue increasing their stroke frequency to gain more speed by using “straight pull“ arm movement pattern [49]. Therefore, compromising the propulsion efficiency to gain extra speed, it seems that particular swimming disciplines are subjected to specific sports evolutionary processes and the search is on for the way to optimize the selection of sprint swimmers, which are consequentlly taller, have less body fat and have more contractile potential.

According to the defined gender performance predictive models (Table 3), body composition elements may represent biological potential for the achievement of better sprint swimming performance. Relation between achieved real performance and estimates by defined models of prediction, showed us that both models underestimate real performance, but much more so in males than in females (intercept for male is 435.143, and for female is 181.114, Figure 1 and Figure 2). Other possible explanations could be connected with a sample structure by stroke, as the majority of swimmers is free-stylers (39.0%), and then breast-strokers (24.4%) and back and fly strokers (by 17.5%, respectively). It was established that, for well trained competitive swimmers, freestyle (front crawl) is the most economic stroke among competitive swimming strokes, followed by the backstroke, the butterfly, and the breaststroke [17,38]. At the same time, swimming forces that can be produced during the 30 s maximal intensity tethered swimming conditions, showed that a breast-stroker can produce more forcefull strokes, than buter-flyers, front crawlers, and back-strokers [43].

The results of the actual study showed us that body composition related to gender specificity characteristics should be considered much more in the future as one of important issues within swimming science [50] along with the known bio-physical determinants related to swimming as: hydrodynamics, kinematics, energetics, and kinetics characteristics [41,49], anthropo-morphological characteristics [13,19,35,47], strength, and power capabilities [20,37,42,45].

This research identified that sprint performance at the male international swimmers level, first and foremost, considering body composition, is associated in male swimmers with specifically balanced presence of proteins and fats (PFI), as well as the balanced ratio between the total body volume and the percentage of fat present in the body (IBC) (Table 1 and Table 2). Secondly, the body predisposition to achieve international level results implies a body composition having a high percentage of muscle mass in relation to all three observed parameters: the absolute value (SMM), along with relative value, which is independent of the body volume (PSMM), and body length (SMM). The results have shown that the variables used to define the mass component of the body had a negative but statistically insignificant correlation and, thus, were also without a predictive potential with regards to the sprint-swimmers performance. This is most likely the consequence of the average values of fat percentage in male swimmers being low (PBF = 9.82 ± 3.35 %, Table 1) along with sprint swimmers with higher PBF values having inferior spring performances, and vice versa (Table 1 and Table 2).

On the other hand, the body predisposition in female swimmers aiming at international level performance presumes in the first place a high percentage of muscle mass (both for relative and absolute values), and in the second place it presumes high value presence of fat-free body mass (PSMM, SMM, FFM, and PFFM) (Table 1 and Table 2).

All the investigated body mass variables, regardless of the specialization method used (relative - PBF, longitudinal independent -FMI, and the absolute -BF), have had a statistically significant and negative correlation with the sprinting performance. In other words, a direct inversely proportional relation has been established between the body mass and the performance of female sprint swimmers, as those with higher PBF values had inferior results, and vice versa (Table 1 and Table 2).

Larger multiseason longitudinal cohort study, with body composition measurement in different training periods, would provide new further evidence to assess in-season and off-season specific relations between body characteristics of swimmers and performance, and not only in sprinters, but also in middle and long distance specialists. Moreover, in future research, it is necessary to determine the relationships between body composition and swimming performance in relation to different swimming techniques, which is one of the limiting factors of this study.

## 5. Conclusions

The current study presents several important and significant correlations between body composition variables and sprint swimming performance in male and female international level sprint swimmers. Generally, quantifying body composition is beneficial for sprint swimmers in order to control the process of training to improve performance. Body composition measurement procedures may be helpful to determine optimal training modalities (methods, volume, and intensity) for continuous improvement of sprint performance.

Considering body composition, sprint-swimming enhanced performance in males is significantly associated with optimal balance between contractile and noncontractile tissue; and with optimally high level of muscle tissue, while for females, optimally high level of muscle tissue with proper low level of fat is primary determinant.

According to MRA results, it could be concluded that it is possible to predict swimming performance in sprint swimmers by body composition variables. Defined models explained 35.1 and 75.1% of the mutual variability of performance, with standard error of 57.48 and 55.99 FINA score, for male and female swimmers, respectively.

This data clearly demonstrates the necessity to monitor body composition characteristics in sprint swimmers, at the international level, even during the preparation training season, as well as during the competition period. This provides a valuable system of collecting information for coaches. Generally speaking, body composition control in international sprint swimmers should be a valuable system of control of the efficiency of body adaptation on training process aimed at optimizing competitive performance potential.

## Figures and Tables

**Table 1:** Descriptive statistics for male and female swimmers.

Variables	Male													
	BH	BM	BMI	BF	SMM	FFM	PBF	PSMM	PFFM	FMI	SMMI	FFMI	IBC	PFI
Mean	186.3	82.4	23.73	8.12	43.13	74.29	9.82	52.36	90.18	2.35	12.41	21.38	2.80	2.18
Std. Dev.	5.4	6.5	1.35	3.08	3.55	6.25	3.35	1.83	3.35	0.88	0.63	1.17	1.25	1.08
cV%	2.90	7.83	5.69	37.07	8.23	8.41	33.81	3.50	3.71	36.17	5.08	5.47	43.21	47.25
SEM	0.78	0.95	0.20	0.37	0.52	0.92	0.41	0.27	0.49	0.09	0.09	0.17	4.29	4.59
SEM (%)	0.42	1.15	0.84	4.56	1.21	1.24	4.18	0.52	0.54	3.83	0.73	0.80	4.29	4.59
Min	178.4	71.8	21.42	2.4	36.2	63.1	2.99	48.44	81.14	0.68	10.95	18.85	1.34	0.89
Max	201.5	96.9	27.13	17.8	54.0	93.1	18.86	55.79	97.01	4.79	13.92	23.86	7.66	6.46
KSZ	0.942	0.770	0.542	1.066	0.756	0.678	0.777	0.632	0.765	1.128	0.554	0.428	1.384	1.380
KS *p*	0.338	0.594	0.931	0.206	0.616	0.747	0.581	0.820	0.603	0.157	0.919	0.993	0.051	0.052
	**Female**													
Mean	173.4	62.8	20.88	9.87	29.52	52.93	15.79	47.01	84.27	3.31	9.80	17.56	1.48	1.21
Std. Dev.	5.8	4.9	1.13	3.01	2.30	5.18	4.84	2.93	4.83	1.11	0.58	0.99	0.48	0.51
cV%	3.36	7.79	5.41	30.50	7.79	9.79	30.65	6.23	5.73	33.53	5.92	5.64	32.43	38.84
SEM	0.97	0.82	0.19	0.40	0.50	0.86	0.78	0.49	0.81	0.16	0.10	0.17	0.07	0.07
SEM (%)	0.56	1.31	0.91	4.05	1.69	1.62	4.94	1.04	0.96	4.83	1.02	0.97	4.73	5.79
Min.	163.0	53.8	19.25	4.4	22.3	41.0	7.50	39.05	70.09	1.47	8.39	15.24	0.73	0.46
Max.	184.4	73.3	23.94	17.5	35.0	62.4	29.91	52.14	92.48	6.51	11.22	20.24	2.61	2.45
KSZ	0.642	0.861	1.052	0.781	0.600	0.630	0.706	0.737	0.710	0.861	0.597	0.589	1.456	1.289
KS *p*	0.804	0.448	0.218	0.575	0.864	0.823	0.702	.649	0.695	0.449	0.868	0.879	0.037	0.072

**Table 2:** Correlation matrix for body composition variables and athletes’ performance with Fisher r-to-z transformation results.

Body Composition Variables		FINA Score Pearsons Correlation Coefficient		Fisher r-to-z Transformation	*p*
		Male	Female		
BH (cm)	r value	0.187	0.535	−1.76	0.078
	*p* significance	0.212	0.001		
BM (kg)	r value	0.215	0.396	−0.87	0.384
	*p* significance	0.151	0.017		
BMI (kg·m^−2^)	r value	0.087	−0.085	0.75	0.453
	*p* significance	0.566	0.621		
BF (kg)	r value	−0.148	−0.566	2.13	0.033
	*p* significance	0.326	0.000		
SMM (kg)	r value	0.350	0.730	−2.43	0.015
	*p* significance	0.017	0.000		
FFM (kg)	r value	0.294	0.702	−2.61	0.009
	*p* significance	0.047	0.000		
PBF (%)	r value	−0.224	−0.695	2.72	0.007
	*p* significance	0.135	0.000		
PSMM (%)	r value	0.353	0.732	−2.44	0.015
	*p* significance	0.016	0.000		
PFFM (%)	r value	0.223	0.697	−2.74	0.006
	*p* significance	0.136	0.000		
FMI (kg·m^−2^)	r value	−0.170	−0.642	2.55	0.011
	*p* significance	0.260	0.000		
SMMI (kg·m^−2^)	r value	0.323	0.684	−2.17	0.030
	*p* significance	0.029	0.000		
FFMI (kg·m^−2^)	r value	0.228	0.621	−2.14	0.032
	*p* significance	0.127	0.000		
IBC (Arbitraly Unit)	r value	0.391	0.687	−1.85	0.064
	*p* significance	0.007	0.000		
PFI (kg)	r value	0.392	0.655	−1.60	0.110
	*p* significance	0.007	0.000		

**Table 3:** Multivariate Regression Analysis (MRA) statistics results with the multiple equation models of athletes’ performance prediction by body composition characteristics—Model Summary.

Model	Dependent Variable	Predictors (Variable, t And *p* Values)	R	R^2^	Adj. R^2^	SEE	ANOVA	
							F Relation	*p* Value
Male	FINA_Score	PFI (1.58, 0.123), BM (2.28, 0.028), PSMM (2.03, 0.049), BH (−2.20, 0.034), IBC (−1.56, 0.126), SMMI (−1.72, 0.093)	0.662	0.438	0.351	57.48	5.06	0.001
Female	FINA_Score	PFI (2.66, 0.013), BM (2.19, 0.037), BMI (−2.28, 0.030), FFMI (2.50, 0.019), PSMM (2.88, 0.008), IBC (−2.60, 0.015), PBF (3.00, 0.006)	0.895	0.801	0.751	55.99	16.10	0.000
**Multiple equation models of athletes’ performance prediction by body composition characteristics for male and female swimmers**								
Male	FINA score_M = 5884.616 − (BH × 65.548) + (BM × 74.835) + (PSMM × 116.793) − (SMMI × 411.608) − (IBC × 268.620) + (PFI • 316.588)							
Female	FINA score_F = −12241.319 + (BM × 6.225) − (BMI × 716.712) + (PBF × 272.470) + (PSMM × 111.570) + (FFMI × 1051.171) − (IBC × 2253.208) + (PFI × 2359.262)							
